# Protocol for transformation-free genome editing in plants using RNA virus vectors for CRISPR-Cas delivery

**DOI:** 10.1016/j.xpro.2024.103437

**Published:** 2024-11-05

**Authors:** Huanhuan Lou, Haiying Xiang, Wanli Zeng, Jiarui Jiang, Jianduo Zhang, Li Xu, Chenglu Zhao, Qian Gao, Zhenghe Li

**Affiliations:** 1 Institute of Biotechnology, Zhejiang University, Hangzhou, Zhejiang 310058, China; 2 Yunnan Academy of Tobacco Science, Kunming, Yunnan 650106, China

**Keywords:** plant sciences, CRISPR, biotechnology and bioengineering

## Abstract

Plant virus vectors have emerged as promising tools for CRISPR-Cas reagent delivery. Here, we present a protocol for DNA-free plant genome editing using an engineered RNA virus vector for the transient delivery of CRISPR-Cas components. We describe steps for viral vector construction, viral vector recovery through agroinoculation of *Nicotiana benthamiana*, mechanical inoculation of target plant hosts, analysis of somatic mutagenesis frequency, and regeneration of mutant plants. The method achieves high editing efficiency and eliminates the need for stable plant transformation.

For complete details on the use and execution of this protocol, please refer to Liu et al.^1^

## Highlights

• Protocol for plant genome editing using TSWV-delivered CRISPR-Cas nuclease
• Instruction on the construction and recovery of TSWV vector
• Steps for inoculation of plants with TSWV vectors
• Procedure for regenerating mutant plant *in vivo* via tissue culture

## Abstract (editor highlights)

Publisher’s note: Undertaking any experimental protocol requires adherence to local institutional guidelines for laboratory safety and ethics.

## Graphical abstract

**Figure:**
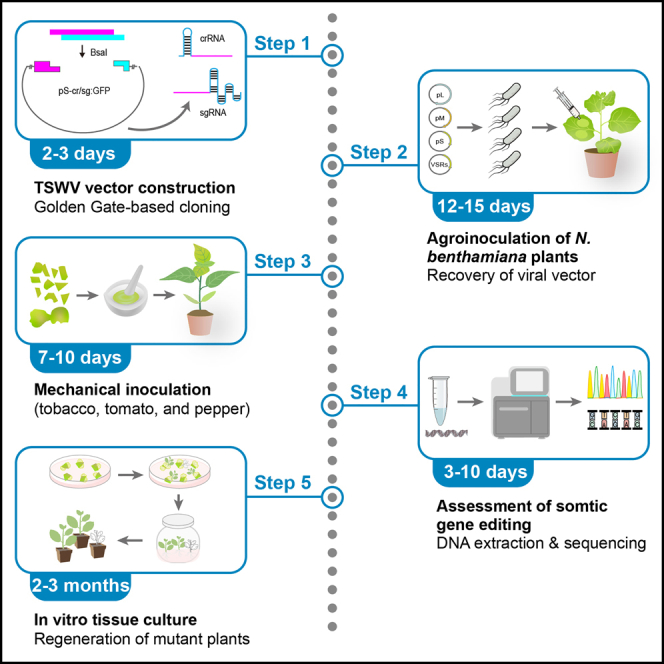

## Before you begin

Traditional plant genome editing has primarily depended on transgene-based delivery of CRISPR/Cas components.^2^ Recently, several plant virus vectors have emerged as effective tools for the transient delivery of CRISPR/Cas through the infections of intact plants.^3^^,^^4^^,^^5^ The advantages of viral delivery methods include simplicity, speed, and robustness. Among these vectors, negative-stranded RNA viruses like Sonchus yellow net virus and tomato spotted wilt virus (TSWV) are particularly notable for their large carrying capacities. These capacities allow for the delivery of entire CRISPR/Cas components or even larger base editors, thus circumventing the need for stable transformation.^1^^,^^6^ Here, we present a procedure for plant genome editing utilizing TSWV-based CRISPR/Cas delivery systems, which has demonstrated effectiveness across multiple plant species, including common tobacco (*Nicotiana tabacum*), tomato (*Solanum lycopersicum*), peppers (*Capsicum annuum*, *C. chinense*, and *C. frutescens*), ground cherry (*Physalis alkekengi*), and peanut (*Arachis hypogaea*), as well as various cultivars of the crop hosts.^1^

**Figure 1:**
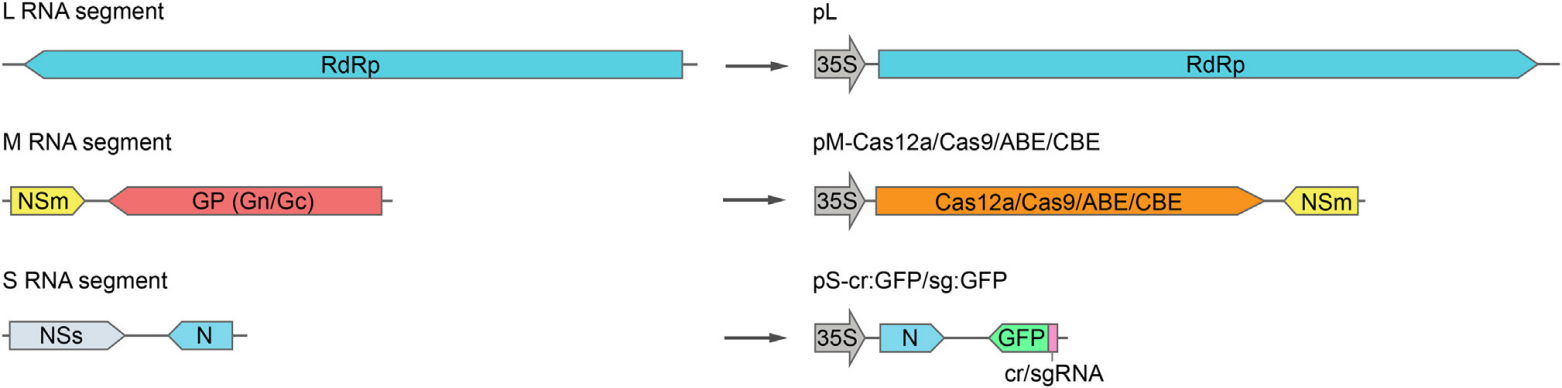
Schematic representation of tomato spotted wilt virus (TSWV) genome organization (left) and viral vectors for the delivery of CRISPR/Cas components (right) Note that the three genome segments (L, M, and S) are shown in negative-sense orientation, while the viral constructs express positive-sense antigenomic RNA derivatives driven by the 35S promoter. 35S, cauliflower mosaic virus 35S promoter; RdRp, RNA-dependent RNA polymerase; NSm, nonstrucutral protein M, NSm; GP, glycoprotein precursor; NSs, nonstructural protein S; N, nucleocapsid protein; GFP: green fluorescent protein; crRNA: Cas12a CRISPR RNA; sgRNA: Cas9 single guide RNA.

TSWV is a trisegmented, negative-strand RNA virus consisting of large (L), medium (M), and small (S) genomes that employ either negative-sense (L) or ambisense (M and S) coding strategies.^7^ The TSWV vectors presented here are composed of three binary constructs: (i) the pL plasmid, which encodes the L genomic RNA, (ii) one of the M genome-based plasmids, which contains an inserted gene encoding either Cas9 (pM-Cas9), Cas12a (pM-Cas12), an adenine base editor (pM-ABE), or a cytosine base editor (pM-CBE) in place of the viral glycoprotein (*G*) gene, and (iii) one of the S genome-based plasmids, which includes the coding sequence for green fluorescent protein (GFP) fused to either Cas9’s single guide RNA (pS-sg:GFP) or Cas12a′s CRISPR RNA (pS-cr:GFP) in place of the viral *NSs* gene (Figure 1). To target a specific gene locus for mutagenesis or base editing, researchers only need to create a pS-based construct that encodes a guide RNA with a customized protospacer sequence.

To initiate recombinant TSWV infection, these viral constructs, along with three binary plasmids designed to express viral suppressors of RNA silencing (VSRs),^8^ namely, pGD-p19, pGD-P1/Hc-Pro, and pGD-γb, are separately transformed into *Agrobacterium tumefaciens* cells and co-delivered into the leaves of *N. benthamiana* plants through agroinfiltration. The use of VSRs and the model plant *N. benthamiana* ensures high-efficiency *Agrobacterium*-mediated transient expression, which is critical for the recovery of recombinant TSWV, as well as other negative-strand RNA viruses of plants.^9^ Although direct agroinoculation of other plant species has not been feasible, leaf sap extracted from infected *N. benthamiana* can be used as an inoculum to transmit the recovered TSWV vectors to additional host plants via mechanical inoculation. Once systemic infection occurs in these plants, the TSWV vectors express a Cas effector protein and guide RNA to induce targeted gene mutagenesis in infected somatic tissues. Subsequently, infected tissues are cultured *in vitro* to regenerate plants with germ line-transmissible mutations. The tissue culture media contain no antibiotic selection; rather, the antiviral compound ribavirin may be supplemented to eliminate the TSWV vectors in regenerated plants and to enhance the recovery of plants with heritable editing.

This protocol encompasses the Golden Gate assembly of a target site-specific protospacer sequence into the viral construct, the recovery of recombinant viral vectors via agroinoculation of *N. benthamiana* plants, sap inoculation of other host plants including tobacco, tomato, and pepper, analysis of somatic editing frequency in virus-infected plants, and regeneration of genome-edited tomato plants from virus-infected tissues.

### Institutional permissions and biosafety considerations

It is important to note that TSWV is classified as a quarantine pest in certain countries and regions, including China and the European and Mediterranean Plant Protection Organization (EPPO). Therefore, before proceeding with this protocol, it is advisable to consult the relevant government agency to determine whether special permission is needed to work with this virus vector.

The TSWV vectors utilized in this procedure lack the essential elements (*G* and *NSs* genes) required for transmission by the insect vector thrips, making them biocontainable and significantly reducing agronomical and environmental risks.^1^ Nonetheless, researchers must adhere to governmental and institutional regulations regarding work with infectious plant pathogens and recombinant biological materials.

### Plants cultivation and growth conditions

**Timing: 4–5 weeks**

*N. benthamiana* seeds should be sown 4–5 weeks in advance. Steps 1–4 are done in insect-proof growth chambers at 25°C under long-day condition (16 h of light and 8 h of darkness).

1. Sow *N. benthamiana* seeds in a plant growth pot filled with well-moistened seed-starting compost mix for germination. Cover the pot with cling film with breathable holes and place it in a growth chamber.

**CRITICAL:** Avoid overwatering the seedling when the soil is adequately moist.

2. Ten days after sowing, transplant the *N. benthamiana* seedlings into pots filled with a soil mixture (vermiculite: loam: quartz sand in a 9:3:1 ratio). Initially, water the plants once with a water-soluble fertilizer at a concentration of 1 g per liter in tap water, and thereafter, maintain soil moisture by watering with tap water every 3–5 days.
3. After 3–4 weeks of cultivation, *N. benthamiana* plants at the 6-true leaf stage are suitable for agroinfiltration.

**CRITICAL:** Only use healthy, vigorously growing plants for agroinoculation to ensure optimal infection efficiency and virus accumulation. It is essential to maintain a well-conditioned growth facility and avoid stressing the plants through excessive watering or exposure to other biotic or abiotic stresses.

***Note:*** The growth duration of *N. benthamiana* may vary based on environmental conditions, so the cultivation time should be adjusted according to the plant’s growth status.

4. Sow the seeds of other crop host plants (e.g., tobacco, tomato, and pepper) for mechanical inoculation.

***Note:*** Coordinating the timing for sowing seeds is crucial to align the inoculation stages of these crop plants with the infection stage of agroinoculated *N. benthamiana*. Generally, pepper seeds take about 15 days, tobacco seeds about 25 days, and tomato seeds about 30 days to reach a suitable size for mechanical inoculation. *N. benthamiana* plants typically show extensive systemic symptoms approximately ten days after agroinoculation, at which point infected leaves can be collected to prepare saps for mechanical inoculation. However, the grow rate of plants can vary based on environmental conditions, so empirical adjustments to the timing may be necessary.

## Key resources table

**Table:** 

REAGENT or RESOURCE	SOURCE	IDENTIFIER
**Antibodies**		

FLAG monoclonal antibody dilution: 1/5,000	Sigma-Aldrich	F1804-50UG
GFP monoclonal antibody dilution: 1/5,000	Abcam	ab32146
HRP-conjugated goat-anti-mouse secondary antibody dilution: 1/10,000	HUABIO, China	HA1006

**Bacterial and virus strains**		

*Escherichia coli* DH5α	N/A	N/A
*A*. *tumefaciens* EHA105	N/A	N/A

**Chemicals, peptides, and recombinant proteins**		

Kanamycin	Sigma-Aldrich	K1377
Rifampicin	Sigma-Aldrich	R3501
Ribavirin	Solarbio	R8370
2-(N-Morpholino) ethanesulfonic acid (MES) monohydrate	BBI Life Sciences	A610341
Acetosyringone	Yeasen	60326ES03
Magnesium chloride hexahydrate (MgCl_2_·6H_2_O)	Macklin	M813913
Mercuric chloride (HgCl_2_)	Thermo Fisher Scientific	LC165909
Dimethyl sulfoxide (DMSO)	Sigma-Aldrich	D5879
Ethylenediaminetetraacetic acid (EDTA)	Sangon Biotech	A500895
Sodium acetate (NaAc)	Sinopharm	10018718
Tris (hydroxymethyl)aminomethane (Tris base)	Sangon Biotech	A501492
2-mercaptoethanol	Thermo Fisher Scientific	21985-023
Tryptone	Sangon Biotech	A505250
TRIzol	Takara	TKR-9109
Yeast extract	Sangon Biotech	A515245
Sodium chloride (NaCl)	Sinopharm	100193611
Bacteriological agar	Sangon Biotech	A505255
Boric acid	Sinopharm	10004818
Sodium dihydrogen phosphate dihydrate (NaH_2_PO_4_·2H_2_O)	Sinopharm	20040718
Disodium hydrogen phosphate dodecahydrate (Na_2_HPO_4_·12H_2_O)	Sinopharm	10020318
Sodium sulfite anhydrous (Na_2_SO_3_)	Macklin	S818067
Potassium dihydrogen phosphate (KH_2_PO_4_)	Sinopharm	10017618
Murashige & Skoog (MS) salt including vitamins	Duchefa Biochemie	M0222
6-benzylamino purine	Coolaber	CB2611
3-indoleacetic acid	Sigma-Aldrich	I2886
Sucrose	Sangon Biotech	A502792
Glucose	Sigma-Aldrich	G8270
Myo-inositol	Sigma-Aldrich	13011
Phytagel	Sigma-Aldrich	P8169

**Critical commercial assays**		

2×TOROBlue Flash KOD Dye Mix	Torovid Technology Company	KRT-200P
Green Taq Mix	Vazyme	P131-01
*Bsa*I (10 U/μL)	Thermo Fisher Scientific	ER0291
2 × Rapid Taq Master Mix	Vazyme	P222-03
Annealing buffer for DNA oligos (5×)	Beyotime Biotechnology	D0251
T4 DNA ligase	Thermo Fisher Scientific	EL0014
FastPure gel DNA extraction mini kit	Vazyme	DC301-01
FastPure plasmid mini kit	Vazyme	DC201-01
TransScript one-step RT-PCR SuperMix	TransGen	AT411-02
Celite 545	Thermo Fisher Scientific	68855-54-9
Carborundum 320 grit	Thermo Fisher Scientific	409-21-2

**Experimental models: Organisms/strains**		

*N*. *benthamiana*	N/A	N/A
*N*. *tabacum* cv. K326	N/A	N/A
*Solanum lycopersicum* cv. Zhongshu-4	N/A	N/A
*Capsicum annuum* cv. Zunla-1	N/A	N/A

**Recombinant DNA**		

pL	Liu et al.^1^	Addgen plasmid #196286
pM-Cas9	Liu et al.^1^	Addgen plasmid #196325
pM-Cas12a	Liu et al.^1^	Addgen plasmid #196291
pM-ABE	Liu et al.^1^	Addgen plasmid #196292
pM-CBE	Liu et al.^1^	Addgen plasmid #196293
pS-sg:GFP	Liu et al.^1^	Addgen plasmid #196296
pS-cr:GFP	Liu et al.^1^	Addgen plasmid #196295
pGD-p19	Ganesan et al.^8^	Addgen plasmid #196326
pGD-γb	Ganesan et al.^8^	Addgen plasmid #196327
pGD-P1/Hc-Pro	Ganesan et al.^8^	Addgen plasmid #196328

**Oligonucleotides**		

Primer: Nos/F: CGGCAACAGGATTCAATCTTAAG	Liu et al.^1^	N/A
Primer: N/F: CTATGGATTACCTCTTGATGATGC	Liu et al.^1^	N/A
Primer: N/R: TTAAGCAAGTTCTGCAAGTTTTG	Liu et al.^1^	N/A
Primer: Actin/F: CAATCCAGACACCTGTACTTTCTCTC	Liu et al.^1^	N/A
Primer: Actin/R AAGCTGCAGGTATCCATGAGACTA	Liu et al.^1^	N/A

**Software and algorithms**		

*Nicotiana benthamiana* Sequencing Consortium	Queensland University of Technology	https://benthgenome.qut.edu.au/
*N*. *tabacum* genome database	Sol Genomics Network	https://solgenomics.net/organism/Nicotiana_tabacum/genome
*Capsicum annuum* genome database	Sol Genomics Network	https://solgenomics.net/organism/Nicotiana_tabacum/genome
CRISPOR	UC Santa Cruz	http://crispor.tefor.net
Inference of CRISPR Edits (ICE)	Synthego	https://ice.synthego.com
Hi-TOM	China National Rice Research Institute	http://www.hi-tom.net/hi-tom/

**Other**		

Spectrophotometer	Thermo Fisher Scientific	ND-1000
Electroporator	Bio-Rad	GenePulser Xcell
Handheld ultraviolet lamp	Analytik Jena US	UVP BLAK-RAY
Fluorometer	Life Technologies	Qubit 2.0
Fluorescence microscope	Zeiss	Lumar V12
Laminar flow hood	Airtech	VS-840K
Fume hood	SunLab	2004-SFH
Steel beads (2 mm)	Longreen	SR60023

N/A, not applicable.

## Materials and equipment

***Note:*** Culture media and buffer solutions should be prepared using MilliQ H_2_O and autoclaved at 121°C for 20 min. Store at 25°C unless otherwise stated.

### Preparation of media

**Timing: 1 day**

**Table:** Lysogenic broth (LB) liquid medium (pH 7.0)

Reagent	Final concentration	Amount
Tryptone	1.0%	10 g
Yeast extract	0.5%	5 g
NaCl	1.0%	10 g
MilliQ H2O	–	Up to 1 L
**Total**	–	**1 L**

***Note:*** Sterilize by autoclaving. Antibiotics can be added as required. LB liquid medium with antibiotics can be stored at 4°C for up to one week. Always handle media under a laminar flow bench to prevent contamination.

LB agar plates: Add 15 g of bacteriological agar powder to the LB liquid medium.

***Note:*** After autoclaving at 121°C for 20 min, allow the media to cool to 55°C. Add antibiotics, mix thoroughly, and pour into sterile petri plates under a laminar flow bench. Let the agar solidify, then store the plates inverted at 4°C for up to one week.

**Table:** Yeast Extract and Peptone (YEP) liquid medium (pH 7.0)

Reagent	Final concentration	Amount
Tryptone	1.0%	10 g
Yeast extract	1.0%	10 g
NaCl	0.5%	5 g
MilliQ H_2_O	–	Up to 1 L
**Total**	–	**1 L**

***Note:*** Sterilization, storage, and adding antibiotics for YEP liquid media may follow the same procedures as for LB media.

YEP agar plates: Add 15 g of bacteriological agar powder to the YEP liquid medium.

***Note:*** The process for preparing YEP agar plates is similar to that for LB agar plates.

**Table:** MS induction medium

Reagent	Final concentration	Amount
MS salts	4.4 g/L	4.4 g
Sucrose	30 g/L	30 g
6-benzylaminopurine	2 mg/L	2 mg
3-Indoleacetic acid	0.1 mg/L	0.1 mg
MilliQ H_2_O	–	Up to 1 L
**Total**	–	**1 L**

***Note:*** Adjust pH to 5.8, add 4 g of phytagel, and autoclaved at 121°C for 20 min. Allow the medium to cool to approximately 50°C and then proceed to pour into the plates.

MS induction medium supplemented with ribavirin: After autoclaving, allow the MS induction medium to cool to approximately 50°C. Then, add 1 mL of a 40 mg/mL ribavirin solution, mix thoroughly by gently shaking, and pour into sterile petri plates under a laminar flow bench.

**Table:** MS rooting medium

Reagent	Final concentration	Amount
MS salts	4.4 g/L	4.4 g
Sucrose	10 g/L	10 g
Myo-inositol	100 mg/L	100 mg
Indole-3-acetic acid	2 mg/L	2 mg
MilliQ H_2_O	–	Up to 1 L
**Total**	–	**1 L**

***Note:*** Adjust pH to 6.0, add 4 g of phytagel, and autoclaved at 121°C for 20 min. Allow the medium to cool to approximately 50°C and then proceed to pour into the plates.

### Preparation of buffer

**Timing: 1 day**

Kanamycin (50 mg/mL): Dissolve 0.5 g of kanamycin powder in 10 mL of MilliQ H_2_O, sterilize through a 0.22 μm filter, and store in 1-mL aliquots at −20°C, for up to 1 year. Dilute stock to 50 μg/mL in specified culture media.

Rifampicin (50 mg/mL): Dissolve 0.5 g of rifampicin powder in 10 mL of DMSO, sterilize through a 0.22 μm filter, and store in 1-mL aliquots at −20°C in the dark, for up to 6 months. Dilute stock to 50 μg/mL in specified culture media.

Ribavirin (40 mg/mL): Dissolve 0.4 g of ribavirin powder in 10 mL of MilliQ H_2_O, sterilize through a 0.22 μm filter, and store in 1-mL aliquots at −20°C in the dark, for up to one year. Dilute stock to 40 μg/mL in specified MS induction medium.

MES pH 5.6 (100 mM): Dissolve 21.325 g of MES monohydrate powder in 900 mL of MilliQ H_2_O, adjust pH to 5.6 with KOH, take the volume to 1000 mL, and sterilize through a 0.22 μm filter. Store at 4°C in the dark, for up to 6 months.

MgCl_2_ (1 M): Dissolve 20.33 g of MgCl_2_·6H2O in MilliQ H_2_O to a final volume of 100 mL, and sterilize through a 0.22 μm filter. Store for up to 1 year at room temperature.

Acetosyringone (100 mM): Dissolve 9.81 g of acetosyringone powder in 500 mL of DMSO, and store in single-use 500 μL aliquots at −20°C in the dark, for up to 6 months.

NaH_2_PO_4_ (0.2 M): Dissolve 31.2 g of NaH_2_PO_4_·2H_2_O in MilliQ H_2_O to a final volume of 1 L. Store at 25°C.

Na_2_HPO_4_ (0.2 M): Dissolve 71.6 g of Na_2_HPO_4_·12H_2_O in MilliQ H_2_O to a final volume of 1 L. Store at 25°C.

NaAc (3 M): Dissolve 40.8 g of NaAc·3H_2_O powder in 40 mL of MilliQ H_2_O, adjust with acetic acid pH to 5.2, adjust the final volume up to 100 mL, and sterilize by autoclaving and store at 25°C.

EDTA pH 8.0 (0.5 M): Dissolve 146.12 g of EDTA in 800 mL of MilliQ H_2_O, adjust pH to 8.0 with NaOH, take the volume up to 1 L. Store at 25°C.

***Note:*** When the pH reaches 8.0, EDTA can dissolve completely.

Tris-HCl pH 8.0 (1 M): Dissolve 121.1 g of Tris powder in 800 mL of MilliQ H_2_O, adjust pH to 8.0 with concentrated HCl (approximately 42 mL), take the volume up to 1 L, sterilize by autoclaving and store at 25°C.

HgCl_2_ (0.1%, w/v): Dissolve 0.1 g of HgCl_2_ powder in 100 mL of MilliQ H_2_O. Store at 25°C in the dark in a secured place.

**CRITICAL:** HgCl_2_ is a highly toxic compound that volatizes slightly at ordinary temperature and appreciably at 100°C. Do not handle until all safety precautions have been read and understood. Prepare the solution under a fume hood and use personal protective equipment as required.

**Table:** Infiltration buffer

Reagent	Final concentration	Amount
MES pH 5.6 (100 mM)	10 mM	50 mL
MgCl_2_ (1 M)	10 mM	5 mL
Acetosyringone (100 mM)	100 μM	500 μL
MilliQ H_2_O	–	Up to 500 mL
**Total**	–	**500 mL**

***Note:*** Freshly prepared before use.

**Table:** 0.1 M potassium phosphate (pH 7.0)

Reagent	Final concentration	Amount
NaH_2_PO_4_·2H_2_O (0.2 M)	–	195 mL
Na_2_HPO_4_·12H_2_O (0.2 M)	–	305 mL
MilliQ H_2_O	–	500 mL
**Total**	–	**1 L**

**Table:** Inoculation buffer

Reagent	Final concentration	Amount
Potassium phosphate pH 7.0 (0.1 M)	0.1 M	20 mL
Na_2_SO_3_	0.5% (wt/vol)	0.1 g
2-mercaptoethanol	0.1% (vol/vol)	20 μL
**Total**	–	**20 mL**

***Note:*** Freshly prepared before use.

**Table:** CTAB buffer

Reagent	Final concentration	Amount
CTAB	2% (wt/vol)	2 g
1 M Tris-HCl (pH 8.0)	100 mM	10 mL
0.5 M EDTA (pH 8.0)	20 mM	4 mL
NaCl	1.4 M	8.2 g
2-mercaptoethanol	2% (vol/vol)	2 mL
MilliQ H_2_O	–	Up to 100 mL
**Total**	–	**100 mL**

***Note:*** Add 2-mercaptoethanol before use to the solution before use; mix well by shaking.

**Table:** TE buffer

Reagent	Final concentration	Amount
1 M Tris-HCl (pH 8.0)	10 mM	1 mL
0.5 M EDTA (pH 8.0)	1 mM	0.2 mL
MilliQ H_2_O	–	98.8 mL
**Total**	–	**100 mL**

**Table:** 5× TBE buffer

Reagent	Final concentration	Amount
Tris-base	445.8 mM	54 g
Boric acid	444.7 mM	27.5 g
0.5 M EDTA (pH 8.0)	10 mM	20 mL
MilliQ H_2_O	–	Up to 1 L
**Total**	–	**1 L**

## Step-by-step method details

### Golden Gate assembly of viral vectors

**Timing: 3–5 days**

**Figure 2:**
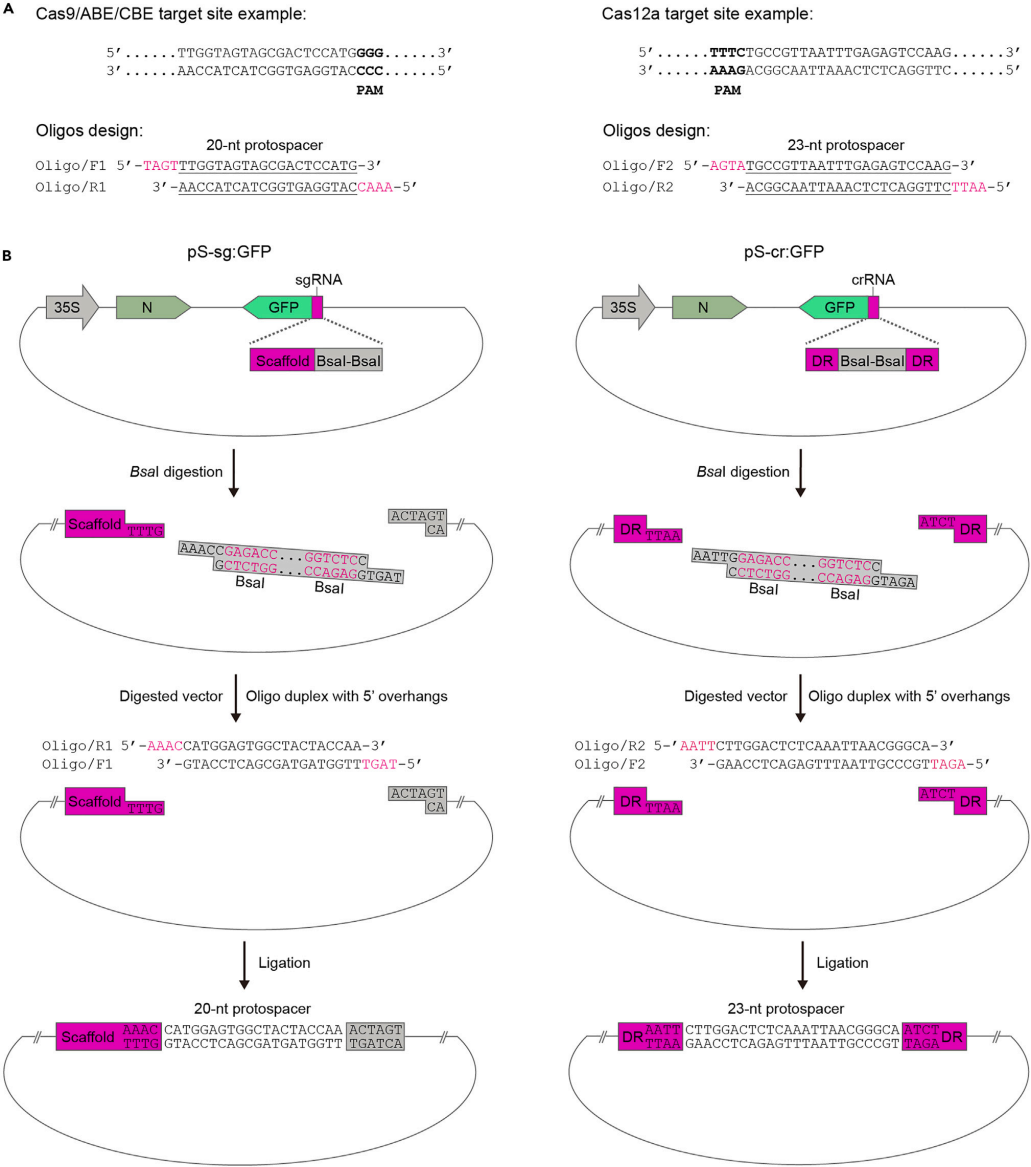
Golden Gate-based assembly of the pS-sg:GFP/cr:GFP constructs for targeting specific gene locus (A) Examples of designing target locus-specific oligonucleotides. Protospacer adjacent motifs (PAMs) in the target sites are shown in boldfaced letters, the protospacer sequences and the appended 5′ *Bsa*I adapter sequences in the designed oligonucleotides are underlined and labeled in magenta, respectively. (B) Schematic representation of Golden Gate cloning steps. sgRNA, single guide RNA; crRNA, CRISPR RNA; scaffold, sgRNA scaffold sequence; DR, crRNA direct repeat sequence.

This section describes the process of inserting a target site-specific protospacer sequence into the pS-sg:GFP or pS-cr:GFP constructs. To enable Golden Gate-based cloning, the pS-sg:GFP construct includes two adjacent *Bsa*I sites located upstream of the sgRNA scaffold sequence from *Streptococcus pyogenes* Cas9 (SpCas9). In contrast, the pS-cr:GFP construct contains two adjacent *Bsa*I sites flanked by two direct repeats of CRISPR RNA from *Lachnospiraceae bacterium* Cas12a (LbCas12a). Two complementary oligonucleotides, corresponding to the target site sequence and appended with compatible adapter sequences, are ligated into the *Bsa*I-digested vectors (Figure 2).

***Note:*** Pay attention to the ambisense coding strategy of the TSWV S genome. The gRNA:GFP fusion is encoded in the negative (genomic)-sense orientation of the S genome.

***Note:*** The direct repeats in pS-cr:GFP were engineered to precisely release mature crRNA for LbCas12a from viral mRNAs. In contrast, pS-cr:GFP does not possess a specific mechanism to process sgRNA for SpCas9 from viral transcripts.

**CRITICAL:** The pS-sg:GFP plasmid must be paired with pM-Cas9, pM-ABE, or pM-CBE, whereas the pS-cr:GFP plasmid is specifically associated with pM-Cas12a.

1. Select target site sequences.
  a. Retrieve the genomic DNA and cDNA sequences of the target gene from relevant genome database, e.g., Sol Genomics Network (https://solgenomics.sgn.cornell.edu/).
  b. Access the online server (http://crispor.tefor.net/) for selection of target site candidates.
    i. Paste the cDNA sequence into the online software server.
      ***Note:*** The input sequence is typically not longer than 2,300 bp, usually representing exons.
    ii. Select the reference genome.
    iii. Select “20 bp-NGG-SpCas9, SpCas9-HF1, eSpCas9 1.1” (for Cas9/ABE/CBE) or “TTT (A/C/G)-23 bp-Cas12a (Cpf1)-recommended, 23 bp guides” (for Cas12a) as the Protospacer Adjacent Motif (PAM).
    iv. Select target sites with predicted efficiency >40% (indicated in the columns labeled “Doenchʹ16” and “Mor.-Mateos scores” in the table).
      ***Note:*** It is essential to consider both the predicted efficiency and specificity when selecting target sites. Choose target sites indicated in green on the website (with green, yellow, and red colors representing high, medium, and low specificity, respectively). Select three to four top-ranking target sites to mitigate potential prediction biases.
      ***Note:*** Align the candidate target site sequences with the genomic sequences to confirm that they are not situated at splicing junctions.
2. Design and synthesize two complementary oligonucleotides for a selected target site (Figure 2A).

**CRITICAL:** The forward oligo sequence corresponds to the non-complementary strand (the non-target strand) and is appended with the 5′ terminal “TAGT” for Cas9 target sites or “AGAT” for Cas12a target sites. The reverse oligo sequence matches the complementary strand (the target strand) and includes the 5′ terminal “AAAC” for Cas9 target sites or “AATT” for Cas12a target sites.

3. Anneal oligonucleotides to make double-stranded DNA.
  a. Dissolve lyophilized forward and reverse DNA oligos separately in 10 mM TE buffer (pH 8.0) to prepare 100 μM stocks.
    ***Note:*** Check the amount of DNA provided by the commercial supplier, and add an appropriate volume of Tris buffer to make a 100 μM stock concentration. Heating (up to 94°C) and vortexing will facilitate resuspension.
  b. Set up the annealing reaction in a microcentrifuge tube.

**Table:** 

Reagent	Amount
Annealing buffer for DNA oligos (5×)	4 μL
Forward oligo (100 μM)	2 μL
Reverse oligo (100 μM)	2 μL
MilliQ H_2_O	12 μL
**Total**	**12 μL**
  c. Heat the mixed oligonucleotides to 95°C for 2 min in a water bath or heat block.
  d. Remove the hot block from the heat source, allowing for gradually cooling to room temperature (about 45 min).
    **Pause point:** The resulting product will be in a stable, double-stranded form and can be stored at 4°C or frozen for several months.
4. Digest the pS-sg:GFP or pS-cr:GFP plasmids with *Bsa*I (Figure 2B).
  a. Set up the digestion reaction in a microcentrifuge tube.

**Table:** 

Reagent	Amount
Plasmid DNA	∼2.5 μg
FastDigest buffer (10×)	5 μL
*Bsa*I	2.5 μL
MilliQ H_2_O	Up to 50 μL
**Total**	**50 μL**
  b. Incubate the digestion mixture at 37°C for 1–2 h.
  c. Purify the digested vector fragment using a gel DNA extraction kit. Check the quality and concentration of the recovered fragment by, for example, agarose gel electrophoresis and ultraviolet spectrophotometry.
5. Assembly of the pS-based targeting vector (Figure 2B).
  a. Set up the ligation reaction in a microcentrifuge tube:

**Table:** 

Reagent	Amount
Linearized vector	50–100 ng
Diluted annealed oligos (1:100 dilution)	1 μL
T4 DNA ligase buffer (10×)	1 μL
T4 DNA ligase	1 μL
MilliQ H_2_O	Up to 10 μL
**Total**	**10 μL**
  b. Incubate the mixture at 22°C for 30 min.
  c. Place tubes on ice immediately once the reaction ends.
    **Pause point:** The ligation mixture can be stored at 4°C for several days.
6. Transformation of *E. coli* cells:
  a. Thaw a tube (100 μL) of chemically-competent *E. coli* DH5α cells on ice.
    ***Note:*** Use competent cells having a transformation efficiency of *ca*. 1 × 10^8^ CFU/μg pUC19 plasmid DNA or higher. Conduct the entire procedure on ice to preserve cell competency.
  b. Gently add the ligation product into the thawed *E. coli* DH5α cells. Mix well by tapping the bottom of tube gently with finger, and then incubate on ice for 30 min.
    ***Note:*** Avoid using a pipette to stir.
  c. Heat shock the tube by placing it in a 42°C water bath for 90 s, then place the tube on ice for 3–5 min.
  d. Add 800 μL of LB liquid medium into the transformation tube, incubate the cells at 37°C with vigorous shaking at 220 rpm for 1 h.
  e. Pellet bacterial cells by centrifuging at 8,000 rpm for 2 min, resuspend the pellet in 100 μL of LB liquid medium.
  f. Spread the suspension onto a LB agar plate supplemented with 50 mg/L kanamycin.
  g. Culture at 37°C for 14 h.
    **Pause point:** After colony appearance, plates can be stored at 4°C for 1–3 days.
7. Screen positive colonies using polymerase chain reaction (PCR).
  a. Synthesize a forward primer “Nos/F”, and use it with the target site-specific reverse primers: Oligo/R1 for pS-sg:GFP and Oligo/R2 for pS-cr:GFP (Figure 2A).
    ***Note:*** The forward primer anneals to the Nos terminator sequence of the pS-based vector backbone.
  b. Prepare PCR master mix and aliquot to PCR tubes.

**Table:** 

Reagent	Amount
Nos/F (10 μM)	1 μL
Oligo/R1 or Oligo/R2 (10 μM)	1 μL
2 × Rapid Taq Master Mix	10 μL
MilliQ H_2_O	Up to 20 μL
**Total**	**20 μL**
  c. Pick a single colony with a sterile flat toothpick or pipette tip. Streak on a new LB agar plate with kanamycin and then swirl the remaining bacteria in a PCR tube.
    **CRITICAL:** Avoid picking too large of a colony. Too many bacteria can inhibit PCR reaction or cause non-specific PCR products.
  d. Perform colony PCR using the following program:

**Table:** 

Steps	Temperature	Time	Cycles
Initial Denaturation	95°C	3 min	1
Denaturation	95°C	15 s	25–30 cycles
Annealing	50°C–60°C	15 s	
Extension	72°C	15 s/ kb	
Final extension	72°C	5 min	1
Hold	4°C	∞
  e. Analyze PCR product sizes by agarose gel electrophoresis.
    ***Note:*** The expected amplicon sizes are 268 bp (pS-sg:GFP) and 291 bp (pS-cr:GFP).
8. Inoculate single positive colonies into culture tubes containing 5 mL of LB liquid medium supplemented with 50 μg/mL kanamycin.
9. Incubated the bacterial cultures at 37°C for 14 h with shaking at 220 rpm.
10. Extract the plasmid from the bacterial cultures using a plasmid miniprep kit.

***Note:*** The plasmids are derived from the pCB301 backbone, which is a low copy-number plasmid in *E. coli*. Approximately 3 μg of plasmid can be recovered from 5 mL of overnight culture, which is typically sufficient for *Agrobacterium* transformation and DNA sequencing.

11. Verify the insert sequence with Sanger sequencing.

### Agroinoculation of *N. benthamiana* plants

**Timing: 12–15 days**

In this section, binary viral constructs are co-delivered into the leaves of *N. benthamiana* plants via infiltration with *Agrobacterium* cell suspensions (agroinfiltration), leading to the generation of recombinant TSWV vectors within systemically infected plant tissues.

***Note:*** TSWV infections cannot be directly initiated through agroinoculation in any host species other than *N. benthamiana* based on our testing.

**CRITICAL:** In addition to the pL, pM-Cas12a/Cas9/ABE/CBE, and pS-cr:GFP/sg:GFP constructs, the VSRs constructs pGD-p19, pGD-P1/Hc-Pro, and pGD-γb, must also be co-delivered into *N. benthamiana* plants for the successful recovery of recombinant TSWV vectors. The inclusion of these VSR plasmids increases transient expression and minimize host antiviral defense response,^9^ ensuring a high efficiency of TSWV recovery (up to 100%).^1^ However, using just pGD-p19 may also yield satisfactory results, with a recovery rate of over 80%. Other potent VSRs may also serve the same purpose but has not been tested in the author’s laboratory.

12. Transform individual viral constructs and binary VSR plasmids into *A. tumefaciens* cells.
  a. Thaw *A. tumefaciens* strain EHA105 electrocompetent cells on ice.
  b. Mix 200 ng of plasmid DNA with 100 μL of *A. tumefaciens* cells in a 1.5-mL tube.
  c. Transfer the mixture into a pre-chilled electroporation cuvette.
    ***Note:*** Add the mixture to the side of cuvette and then tap the liquid down to the bottom to eliminate air bubbles.
  d. Set the Bio-Rad electroporator parameters as follows: voltage at 2,500 V, resistance at 200 Ω, capacitance at 25 μFD, with a 2 mm electrode gap.
  e. Place the cuvettes into the electroporator, press the pulse buttons until a beep sounds (may take a few seconds).
    ***Note:*** Make sure that the cuvettes are dry before placing them into the electroporator. If spark occurs across the electrode, use less amount of plasmid DNA or purify the plasmid to remove residual salts.
  f. Add 800 μL LB liquid medium to the cuvettes.
  g. Transfer the mixture into a 1.5-mL sterile tubes, and incubate at 28°C with shaking (220 rpm) for 2 h.
  h. Centrifuge at 8,000 rpm for 2 min to pellet the *A. tumefaciens* cells. Retain 100 μL of the supernatant to resuspend the pellet.
  i. Plate the suspension onto YEP agar plates containing 50 μg/mL kanamycin and 50 μg/mL rifampicin.
  j. Incubate the plates, upside down, at 28°C for 48 h.
    **Pause point:** After colony appearance, plates can be stored for several days at 4°C.
13. Screen positive colonies by PCR using the specific primers mentioned in Step 7.
14. Inoculate positive colonies into 15-mL culture tubes containing 2 mL of YEP medium supplemented with 50 μg/mL kanamycin and 50 μg/mL rifampicin.
15. Incubate the culture overnight at 28°C with shaking (220 rpm).
16. Dilute the culture in fresh YEP medium with antibiotics at a ratio of 1:100.

***Note:*** The dilution ration can be adjusted in case of time constraints, but should not exceed 1:40 to preserve the *A. tumefaciens* vitality.

17. Continue to culture the cells at 28°C for 10–12 h until the optical density 600 (OD_600_) reaches approximately 1.0.

***Optional:*** Mix 700 μL of the culture aliquots with 300 μL of sterilized glycerol (50% vol/vol) in a sterile 1.5-mL tube and store them at −80°C.

18. Spin down the cultures at 4,000 rpm for 10 min and resuspend them in infiltration buffer (see preparation of buffer) at an optical density at 600 nm (OD_600_) of 0.6.
19. Incubate the cells at room temperature for 2–4 h.
20. Mix the individual bacterial cultures containing pL, one of the pM-Cas12a/Cas9/ABE/CBE plasmid, and one of the pS-cr:GFP/sg:GFP plasmids, pGD-p19, pGD-P1/Hc-Pro, and pGD-γb in appropriate volumes.

**Table:** 

Strain	Final concentration (OD_600_)	Amount
pL	0.1	5 mL
pM-Cas12a/Cas9/ABE/CBE	0.4	20 mL
pS-cr:GFP/sg:GFP	0.05	2.5 mL
pGD-p19	0.017	0.83 mL
pGD-P1/Hc-Pro	0.017	0.83 mL
pGD-γb	0.017	0.83 mL
Infiltration buffer	–	2 mL
**Total**	**0.6**	**30 mL**

***Note:*** The total volume of bacterial mixture needed depends on the number of *N. benthamiana* plants to be infiltrated. Approximately 2 mL of bacterial mixture is sufficient to infiltrate a single plant.

***Note:*** When just the pGD-p19 VSR is used, adjust its final OD_600_ to 0.05.

**Figure 3:**
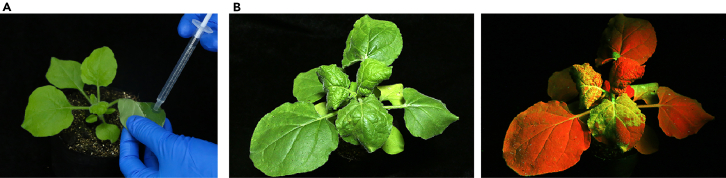
Agroinoculation of *N. benthamiana* plants (A) Image showing agroinfiltration of an *N. benthamiana* plant. (B) Images showing systemic symptoms in agroinoculated plants photographed at 12 days post inoculation under white light (left) and long-wavelength UV illumination (right).

21. Infiltrate the mixed cultures into the abaxial surfaces of fully expanded *N. benthamiana* leaves at approximately 6-true leaf stage using a 1-mL needleless syringe (Figure 3A).

***Note:*** Gently press the syringe to infiltrate the solution into the plant apoplast to minimize leaf damage. Typically, three fully expanded leaves per plant are infiltrated.

22. Transfer the plants to a growth chamber at 25°C and 16-h light/8-h darkness photoperiod.
23. Monitor the symptom appearance of the infiltrated plants (Figure 3B).

***Note:*** Systemic infection of TSWV vectors carrying Cas9 or Cas12a typically appears 7–10 days post infiltration in up to 100% of infiltrated plants, manifested as downward leaf curling, leaf chlorosis, and wilting and necrosis at later stages. For vectors carrying the larger ABE or CBE, there may be a 2–3-day delay in symptom development. However, the onset of disease and infection efficiency can be influenced by growth conditions, such as temperature, humidity, and light intensity and wavelength.

***Optional:*** Successful viral infection can be confirmed by detecting GFP fluorescence using a handheld ultraviolet lamp (e.g., Ultraviolet Products, UK) or a fluorescence microscope, or by conducting reverse transcription-PCR (RT-PCR). If the selected target site is conserved in *N. benthamiana*, somatic gene editing can be assessed at this stage using the method described in Section 4, “analysis of somatic editing frequency”.

**Pause point:** Infected leaf tissues can be stored in −80°C for later use in preparing leaf sap inoculum. However, it is important to note that virus infectivity significantly decreases after freezing and thawing.

### Mechanical inoculation of other host plant species

**Timing: 7–10 days**

TSWV is mechanically transmissible under laboratory conditions.^7^ This section describes the procedure for mechanically inoculating tobacco, tomato, and pepper plants using leaf saps extracted from *N. benthamiana* plants that are systemically infected with the TSWV vectors.

24. Collect 1 g of symptomatic leaf tissues from systemically infected *N. benthamiana* plants.

**Figure 4:**
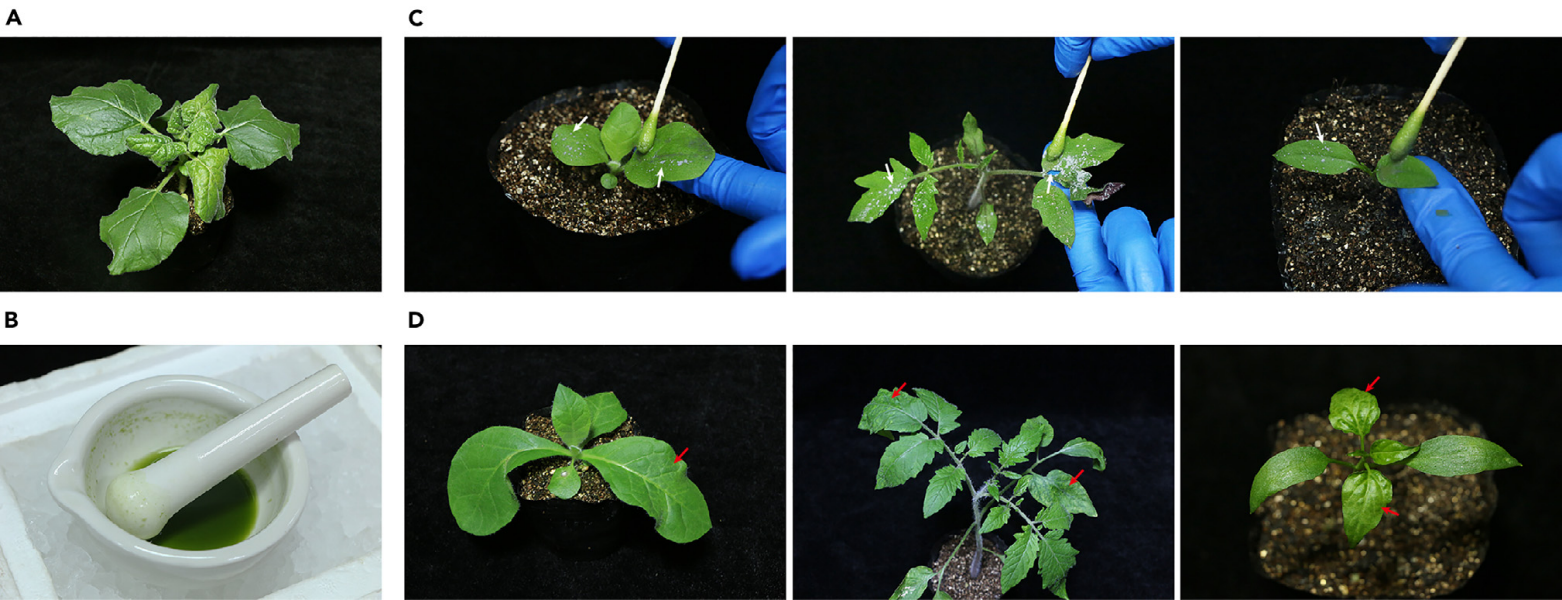
Mechanical inoculation of host plants (A) Image showing an infected *N. benthamiana* plant used for preparing sap inoculum. (B) Images showing leaf sap extracted from the symptomatic leaves displayed in (A) and placed on ice. (C) Images showing mechanical inoculation of tobacco (left), tomato (middle), and pepper (right) plants. White arrows indicate the abrasive mixtures dusted on the leaves to be inoculated. (D) Images showing the leaf symptoms exhibited by the inoculated tobacco (left), tomato (middle), and pepper (right) plants at 9, 9, and 13 days post inoculation, respectively.

**CRITICAL:** Select the leaves exhibiting extensive viral symptoms 3–5 days after the onset of systemic infection (Figure 4A). The symptoms typically persist for about one week before the plants begin to show signs of necrosis and wilting in the leaves, stem, or even growing buds. After this period, newly emerged leaves recover from the viral infections and contain a low concentration of viral particles.

***Alternatives:*** If freshly infected *N. benthamiana* leaf tissues are unavailable when seedlings are ready for inoculation, frozen leaves may be used to prepare the sap inoculum. In this case, it is advisable to inoculate a larger number of plants, as lower infection rates are expected.

25. Homogenize the leaf tissues in 10 mL of ice-cold inoculation buffer using a pre-chilled mortar and pestle.

***Note:*** Ensure the tissues are thoroughly ground to obtain a homogenous mixture. Complete the grinding process within a few min to prevent the buffer and mortar from warming up.

26. Keep the inoculum on ice and perform the inoculation promptly (Figure 4B).

***Note:*** After grinding, virus infectivity decreases over time, even when the sap is kept on ice.

27. Dust the plant leaves with abrasive mixture (carborundum:celite in a 2:1 ratio) (Figure 4C).

***Note:*** Apply the mixture to the first two true leaves of tobacco and tomato, and the two cotyledons of pepper. Avoid excessive application to prevent damage to the leaves.

***Note:*** Ware a face mask to avoid inhaling the abrasives while dusting the plants.

28. Dip a cotton swap in the leaf sap and gently rub the inoculum onto the dusted leaves (Figure 4C).

**CRITICAL:** Apply gentle friction during inoculation to avoid damaging the leaves, while supporting the leaf with the other hand. Always wear gloves when handling plants, and change gloves between different viral inocula to prevent cross-contamination.

29. After inoculation, spray the plants with distilled water within 2–5 min and place them in a growth chamber.
30. Monitor the inoculated plants for the appearance of symptoms.

***Note:*** Approximately 7 days post inoculation, the upper uninoculated leaves will begin to show symptoms, including leaf curling and chlorosis (Figure 4D). As the infection progresses, the infected plants will develop more conspicuous symptoms, such as severe vein clearing, leaf chlorosis, and systemic necrotic lesions.

***Optional:*** Examine the inoculate plants under a fluorescence stereo microscope. GFP fluorescence can be detected in the infiltrated leaves 3–5 days after inoculation. Subsequently, fluorescence will begin to appear in the vascular tissues of systemic leaves and gradually spread into the mesophyll tissues in a few days.

31. Detect virus infection with RT-PCR.
  a. Total plant RNA extraction:
    i. Collect approximately 0.1 *g* of systemically infected leaf tissues in a 2-mL centrifuge tube containing two steel beads.
    ii. Freeze the tube in liquid nitrogen and grind the tissue into powder.
      **Pause point:** After freezing, samples can be stored at −80°C indefinitely.
    iii. Add 1 mL of TRIzol extraction reagent to the powdered tissue, vortex vigorously for 3 min, and keep the mixture at 25°C for 5 min.
      ***Alternatives:*** Other plant total RNA extraction reagents or commercial kits may also be used.
    iv. Add 200 μL of chloroform to the mixture, vortex thoroughly for 15 s, and then incubate at 25°C for 3 min.
    v. Centrifuge the mixture at 12,000 rpm for 15 min at 4°C.
    vi. Carefully transfer the supernatant to a new 1.5 mL RNase-free centrifuge tube.
    vii. Repeat chloroform extraction (step iv) and centrifugation (step v).
    viii. Transfer the supernatant to a new 1.5 mL RNase-free centrifuge tube.
    ix. Add an equal volume of pre-chilled isopropanol, and mix well by inverting the tube several times.
    x. Incubate the mixture at −30°C for 1 h.
    xi. Centrifuge the mixture at 12,000 rpm for 15 min at 4°C.
    xii. Discard the supernatant, and wash the RNA pellet twice with 800 μL of 75% ethanol.
    xiii. Air-dry the RNA pellet in a fume hood for 10 min, and then dissolve it in 50 μL of TE buffer.
    xiv. Measure RNA quality and concentration using a spectrophotometer (e.g., NanoDrop, Thermo Fisher Scientific).
      **Pause point:** Total RNA samples can be used immediately or stored for weeks at −80°C.
  b. RT-PCR detection.
    i. Synthesize a pair of TSWV *N* gene-specific primers N/F and N/R.
    ii. Set up the one-step RT-PCR reaction.

**Table:** 

Reagent	Amount
Total RNA	1 μg
N/F (10 μM)	0.4 μL
N/R (10 μM)	0.4 μL
2 × TransScript One-Step Reaction Mix	10 μL
TransScript One-Step Enzyme Mix	0.4 μL
RNase free water	Up to 20 μL
**Total**	**20 μL**

      ***Note:*** We used TransScript One-Step RT-PCR SuperMix kit; alternative source of reagents may work.
    iii. Set RT-PCR cycling conditions.

**Table:** 

Steps	Temperature	Time	Cycles
Reverse transcription	45°C	15–30 min	1
Initial Denaturation	94°C	25 min	1
Denaturation	94°C	30 s	30–40
Annealing	55°C	30 s	
Extension	72°C	30 s	
Final extension	72°C	5–10 min	1
Hold	4°C	∞
    iv. Analyze PCR products by agarose gel electrophoresis.
      ***Note:*** The expected size of the amplicon is 391 bp. It is recommended to simultaneously amplify an endogenous gene, such as *Actin* using oligos Actin/F and Actin/R, to serve as an internal control.
      ***Optional:*** The integrity of the inserts in progeny virus genomes can be verified by RT-PCR using primers described by Liu et al.^1^ This step is normally not necessary, as TSWV vectors are genetically stable and rarely lose their inserts. However, it can be helpful for troubleshooting in cases where expected gene editing does not occur in the infected tissues.

### Analysis of somatic editing frequency

**Timing: 3–10 days**

This section describes the steps to detect targeted gene editing in the viral vector-infected somatic cells. Here we utilize Sanger sequencing and deep sequencing to determine editing frequency.

32. Collect systemically infected plant tissues 3–5 days after symptom onset.

**Figure 5:**
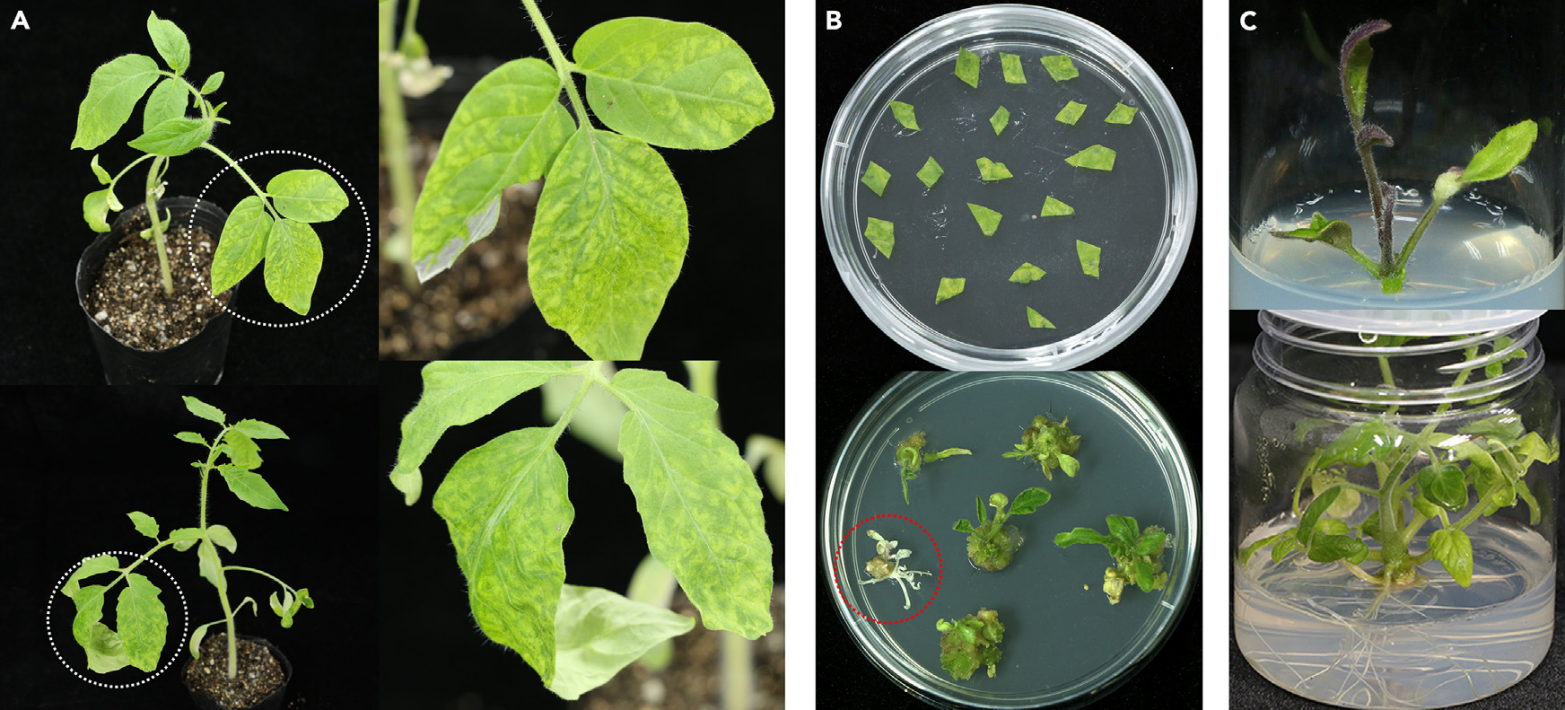
Regeneration of tomato plants from virus-infected leaf tissues (A) Images showing infected tomato plants. White circles indicate systemic leaves with extensive viral symptoms, which are magnified and displayed in the right panels. (B) Upper panel: Dissected tomato leaf explants placed on MS induction medium. Lower panel: Shoots induced from the explants. A red circle marks an albino shoot resulting from the knockout of the *Phytoene Desaturase* (*PDS*) gene. (C) Upper panel: A shoot transferred to MS rooting medium. Lower panel: A shoot with well-developed roots.

***Note:*** Select infected leaves exhibiting well-developed systemic symptoms (Figure 5A). Since CRISPR/Cas gene editing is an accumulative process, leaf tissues 3–5 days after symptom onset can be selected to achieve reasonable high editing efficiencies while avoiding tissue necrosis occurred at the late stages of virus infection.

***Note:*** At the same time, infected leaf tissues are collected for *in vitro* tissue culture for regeneration of gene-edited plants, as described in the following section.

***Optional:*** Total plant protein may be extracted from infected tissues at this stage to detect the abundance of Cas effector and GFP proteins in these tissues. The Cas9, Cas12a, and base editor proteins contain a FLAG tag at their N-termini, facilitating detection by immunoblotting.

33. Extract total DNA from infected plant tissues.
  a. Collect approximately 0.1 *g* of leaf tissues into a 2-mL centrifuge tube containing two steel beads.
    **CRITICAL:** TSWV may not distribute evenly and can form a specific localization pattern in the infected plant tissue, particularly under suboptimal growth conditions for disease progression. Therefore, collect the leaf zones exhibiting extensive symptoms and GFP fluorescence.
    ***Note:*** Include leaf samples collected from wild-type or mock-inoculated plants to serve as negative controls for gene editing assays. Take care to prevent cross-contamination during the extraction process.
    ***Alternatives:*** Other plant total DNA extraction methods or commercial silica column kits may also work.
    **Pause point:** After collection, samples can be stored at −80°C indefinitely.
  b. Freeze the tube in liquid nitrogen and grind the tissue into powder in a homogenizer.
  c. Add 600 μL of CTAB buffer and vortex vigorously.
    ***Note:*** Add 2-mercaptoethanol into the CTAB buffer freshly before use.
  d. Incubate at 65°C for 1 h, and mix every 15 min.
  e. Add 600 μL of a 24:1 mixture of chloroform and isoamyl alcohol, vortex vigorously.
  f. Incubate at 25°C for 10 min.
  g. Centrifuge at 10,000 rpm for 5 min at 4°C.
  h. Transfer the supernatant (approximately 500 μL) to a new 1.5 mL centrifuge tube.
  i. Add an equal volume of pre-chilled isopropanol and 1/10 volume of 3 M NaAc (pH 5.2).
  j. Mix thoroughly and incubate at −30°C for 1 h.
  k. After centrifugation at 13,000 rpm for 10 min at 4°C, discard the supernatant.
  l. Wash the pellet twice with 1 mL of 75% ethanol, followed by centrifugation at 12,000 rpm for 5 min at 4°C each time.
  m. Air-dry the pellet in a fume hood.
  n. Dissolve the pellet in 50 μL of TE buffer.
  o. Measure DNA quality and concentration using a spectrophotometer.
    **Pause point:** DNA samples can be stored for several weeks at −20°C.
34. Analyze somatic mutation frequency by Sanger sequencing.
  a. Design and synthesize target loci-specific oligonucleotides.
    ***Note:*** The recommended amplicon length is 300–500 bp.
    ***Note:*** For targets in polyploid plants with homologous genes (e.g., *N. tabacum*), homolog-specific primers should be used when subjecting PCR products to Sanger sequencing. However, a conserved primer sets can be used for deep sequencing of PCR products.
  b. Set up the amplification mixtures in PCR tubes.

**Table:** 

Reagent	Amount
DNA template	100 ng
Forward primer	1.5 μL
Reverse primer	1.5 μL
2×TOROBlue Flash KOD Dye Mix	25 μL
RNase free water	Up to 50 μL
**Total**	**50 μL**

    **CRITICAL:** Use KOD or alternative high-fidelity DNA polymerases to minimize PCR error rate.
  c. Set PCR cycling condition as follow.

**Table:** 

Steps	Temperature	Time	Cycles
Initial Denaturation	98°C	30 s	1
Denaturation	98°C	10 s	25–30
Annealing	X°C	25 s	
Extension	68°C	10 s/ kb	
Final extension	68°C	5 min	1
Hold	4°C	∞

    ***Note:*** The specific annealing temperature depends on the Tm values of the primers.
  d. Separate PCR products by 1% agarose gel electrophoresis.
  e. Purified PCR products using a Gel DNA Extraction Kit.
    ***Note:*** Dissolve DNA in distilled water instead of TE buffer.
    **CRITICAL:** Ensure that the PCR products are of high quality (single band, no smear) and have sufficient concentration (>100 ng/μL) for sequencing.
  f. Subject the PCR products to Sanger sequencing.
  g. Use the Inference of CRISPR Edits website (ICE) to analyze the editing efficiency.
    ***Alternatives:*** Other online tools may also be used, such as Tracking of Indels by Decomposition (TIDE) (https://tide.nki.nl).
    i. Access the online ICE tool (https://ice.synthego.com), and then click “Analyze my data”.
    ii. Paste the target sequence including the PAM into the “GUIDE SEQUENCE” column.
    iii. Upload a Sanger sequencing file from an uninoculated plant to “CONTROL FILE” and a Sanger sequencing file from an infected plant to “EXPERIMENT FILE”.
    iv. Click “ADD SAMPLE TO ANALYSIS” button, then click “ANALYZE”.
      ***Note:*** Multiple samples can be analyzed simultaneously.
35. Analyze somatic mutation frequency by deep sequencing.
  a. Design and synthesize target loci-specific oligonucleotides with an appended 5′ adapter sequence.
    ***Note:*** The recommended amplification length is 150–300 bp, with a maximum length of 500 bp.
    ***Note:*** The adapter sequences are 5′-GGAGTGAGTACGGTGTGC-3′ (forward primers) and 5′-GAGTTGGATGCTGGATGG-3′ (reverse primers). However, different sequencing facilities may use different adapter sequences, so it is important to confirm the specific requirements with the facility being used.
  b. Amplify the target site sequences using the procedure described in Step 33b-c.
  c. Barcode the PCR products in a second-round amplification using Illumina index primer pairs.
    ***Note:*** The index primers anneal to the universal adapter sequences introduced to the amplicon libraries during the first round of PCR and also contain 5′ appended unique index sequences to barcode each library, allowing multiple libraries to be pooled and sequenced together.
  d. Quantify the products using a fluorometer (e.g., Qubit 2.0, Life Technologies) and dilute it to 1 ng/μL.
  e. Sequence the samples using the Illumina HiSeq platform.
  f. Demultiplex and pre-process paired-end reads to remove adapter sequences and low-quality reads.
  g. Align the clean amplicon sequences to a reference sequence using the Hi-Tom online platform (http://www.hi-tom.net/hi-tom/).^10^
    ***Note:*** We recommend a filter threshold of 1% for target site mutagenesis and 0.1% for base editing.

### Regeneration of mutant plants through tissue culture

**Timing: 2–3 months**

The TSWV vectors do not induce detectable heritable edits in infected plants. In this section, using tomato as an example, we describe the procedure of culturing infected somatic cells *in vitro* to regenerate plants containing heritable mutations, as well as the application of the antiviral compound ribavirin to eliminate the viral vectors from the regenerated plants.

***Note:*** Plant regeneration through tissue culture is a highly species- and genotype-dependent. The following procedure is established with the tomato cultivar Zhongshu-4, and further adjustments to the medium composition and hormone concentrations may be necessary when working with other tomato genotypes or different plant species.

36. Collect infected plant tissues as described in Step 32.

***Note:*** We recommend collecting leaves at 3–5 days after symptom onset (Figure 5A) to optimize the balance between editing efficiency and tissue culture response. However, viral disease progression may be affected by environmental conditions, so empirical adjustments to the timing may be needed.

37. Surface sterilize the leaves by immersing them in a 70% ethanol solution for 20 s.
38. Further sterilize the leaves by immersing them in a 0.1% HgCl_2_ solution for 8 min.

***Note:*** Pay attention to the duration of sterilization. A short sterilizing period may lead to incomplete disinfection, whereas overextended sterilization could damage the leaves and negatively affect subsequent callus induction.

***Note:*** HgCl_2_ is highly toxic and corrosive to mucous membranes. Dispose the used solution in biohazardous containers following local regulations.

39. Immediately rinse the leaves three times in sterile MilliQ water.
40. Remove the main leaf veins and cut the leaf tissues into approximately 1 × 1 cm^2^ pieces using a sterilized scalpel.
41. Place the leaf explants on MS induction medium supplemented with ribavirin, ensuring that the abaxial surfaces are in contact with the medium (Figure 5B, upper panel).
42. Incubate the plates at 25°C under a 16-h light/8-h dark photoperiod for 25 days.

***Note:*** Treatment with 40 mg/L of ribavirin for 25 days resulted in a 100% virus clearance rates in our experiments.^1^ It is possible that lower concentrations of ribavirin and/or a shorter incubation time may also be effective, although this has not yet been evaluated.

43. Transfer the explants to fresh MS induction medium without ribavirin and continue to culture for an additional 20 days.

***Note:*** Ribavirin treatment causes a delay in callus formation and shoot induction. After culturing on ribavirin-free medium, shoots start to emerge in about one week (Figure 5B, lower panel).

44. Excise well-developed shoots from the callus mass and transfer them to MS rooting medium (Figure 5C, upper panel).
45. Continue culture the shoots until their root systems developed adequately (Figure 5C, lower panel). Open the culture bottles and add sterile water to acclimatize the plants for 3–4 days.
46. Transplant the seedlings into soil and cover with plastic film to prevent desiccation. After one week, remove the plastic film, and grow the plants in a greenhouse.
47. Genotype the regenerated plants by Sanger sequencing or deep sequencing, following the procedure described in the earlier section (Steps 33–35).

***Note:*** Our experiments showed that treatment with ribavirin effectively eliminated the viral vectors, enhanced the regeneration of virus-infected cells and improved the recovery of plants with targeted mutations when compared to the mock-treat controls.^1^

## Expected outcomes

Plant viral vectors have emerged as effective tools for delivering genome-editing components in plant research.^3^^,^^4^^,^^5^ In this protocol, we detail the steps for generating recombinant TSWV vectors for the transient delivery of CRISPR/Cas nucleases and base editors across several crop species. The CRISPR/Cas elements are inserted into the tri-segmented viral vector in separate viral genome segments, with the M genome carrying the Cas protein or derivatives and the S genome containing a guide RNA and a fluorescent marker to monitor virus infections. Target gene-specific protospacer sequences can be easily inserted into the S genome construct using the user-friendly *Bsa*I-based cloning method, resulting in viral vectors equipped with custom gRNAs. Once *N. benthamiana* plants are agroinoculated with the viral constructs, recombinant viral vectors can be recovered in 100% of agroinfiltrated plants.

These vectors can be subsequently transmitted to additional plant host species through sap inoculation. Upon systemic infections, the TSWV vectors replicate, spread, and express the CRISPR/Cas components at high levels to enable efficient somatic gene editing in the infected tissues. Additionally, these viral vectors can successfully deliver CRISPR components to different varieties of a given crop host, displaying little host genotype dependency.^1^ Our data showed that high mutagenesis efficiencies, ranging from 51.5% to 78.0%, depending on the target site and host species, were induced by virally delivered CRISPR/Cas12 reagents in tobacco, tomato, peppers, ground cherry, and peanut. Editing frequencies of 63.5% and 78.2% were achieved for the CRISPR/Cas9 nucleases. Furthermore, virally delivered CBE yielded high C:G to T:A conversion frequencies of 29.4%–60.8%, while ABE achieved modest A:T to G:C conversion efficiencies (12.8%–25.2%) in tobacco, tomato, and peppers.^1^

Upon plant regeneration via tissue culture, virus-induced somatic editing can be converted to high rates of germ line-transmissible mutations, particularly when ribavirin treatment is applied to clear the viral vectors, alleviating its negative impact on plant regeneration. In tomato, ribavirin treatment resulted in 68.9% of regenerants containing bi-allelic *PDS* mutations, compared to 40.0% in the mock treatment. For the allotetraploid tobacco, the ratios of biallelic mutations of the two *PDS* homologs were lower, being 30.6% and 16.0% for ribavirin and mock treatments, respectively.^1^

Our recent research demonstrates that bypassing the challenges associated with transgenesis also simplifies the regeneration of plants through tissue culture, enabling efficient recovery of heritable genome editing events, even in recalcitrant species such as pepper.^11^ Collectively, this protocol offers a robust method for non-transgenic genome editing applicable to several plant species.

## Limitations

TSWV has an extremely broad host range, infecting over 1,090 dicotyledonous and monocotyledonous species.^7^ In this protocol, we have only tested several hosts in the Solanaceae family. To expand the application of this viral delivery system to additional host species, further studies combining fundamental virus biology with refined inoculation techniques are required.

Although our study showed that TSWV could infect various varieties of given hosts,^1^ some commercial crop cultivars may contain introgressed resistance genes, such as *Sw-5b* in tomato and *Tsw* in pepper. These cultivars would be unsuitable for the TSWV delivery system.

Like other RNA viruses, TSWV uses its own RNA-dependent RNA polymerase to transcribe inserted foreign genes, producing guide RNA transcripts with extra sequences at both termini. While the type V CRISPR/Cas12a protein has crRNA processing activity, the type II CRISPR/Cas9 lacks a specific mechanism to release sgRNA from a primary transcript and may rely on non-specific host RNases for sgRNA maturation. In the latter case, the availability or activity of mature sgRNA molecules may become a limiting factor.

TSWV causes severe symptoms in many host plants, sometimes even leading to growing bud necrosis and plant death, especially at the late stages of infection.^7^ This severity may limit the time window available for efficient gene editing and regeneration of mutant plants from virus-infected tissues.

Somatic mutations generated in TSWV-infected tissues are not germ line transmissible, necessitating the use of tissue culture to regenerate plants with heritable mutations. However, tissue culture remains a time-consuming and technically challenging process for many plant species and genotypes.

## Troubleshooting

### Problem 1

Few *E. coli* colonies grown or high proportion of empty vectors (step 7).

### Potential solution

• Check the concentration of the digested pS-sg:GFP/cr:GFP vector fragments. The vector backbone is a low-copy plasmid in *E. coli* and difficult to obtain in large quantities. Use a larger volume of *E. coli* culture can help increase the plasmid concentration.
• Analyze the quality of the pS-sg:GFP/cr:GFP minipreps using a spectrophotometer. If necessary, remove impurities using a DNA Cleanup kit or re-purify the plasmid using a Midiprep kit.
• Ensure that the design of oligonucleotides is correct and that the annealing protocol is closely followed. After annealing, the duplex should contain overhangs that match the *Bsa*I-digested vector ends.

### Problem 2

*N. benthamiana* leaves are difficult to infiltrate (Step 21).

### Potential solution

• Infiltrate fully expanded leaves.
• Avoid over-watering the plants and reduce humidity of the growth chamber. Stand the plants on lab bench in a well-ventilated room for 5–10 h prior to infiltration.

### Problem 3

No virus infection in agroinfiltrated *N. benthamiana* plants or the infection rate is low (Step 23).

### Potential solution

• Select *N. benthamiana* plants at the proper leaf stages for infiltration and monitor environmental conditions to prevent environmental stress.
• Grow fresh *A. tumefaciens* colonies.
• Double-check each *A. tumefaciens* strain to confirm the presence of the correct plasmid.
• Ensure the accuracy of the infiltration mixtures by precisely measuring the OD_600_ value of each agrobacterial strains and mixing them in proper ratios.

### Problem 4

Infiltrated leaves develop necrosis, resulting in no systemic viral infection (Step 23).

### Potential solution

• Adjust the plant growth conditions to prevent hypersensitive responses.
• Decrease the OD_600_ value of *A. tumefaciens* strains carrying pGD-p19, pGD-P1/Hc-Pro, and pGD-γb by half (final OD_600_ = 0.0085), or omit pGD-P1/Hc-Pro and pGD-γb.

### Problem 5

Systemic leaves of the infiltrated *N. benthamiana* plants develop only localized infections or rapid necrosis (Step 23).

### Potential solution

• Adjust the plant growth conditions to prevent hypersensitive responses.
• Avoid using sick or stressed plants for infiltration.

### Problem 6

No virus infection in mechanically inoculated plants or the infection rate is low (Step 30).

### Potential solution

• Use seedlings at appropriate ages for inoculation.
• Adjust the growth chamber conditions to create an environment conducive to viral infections.
• Use fresh *N. benthamiana* leaves with well-developed symptoms to prepare sap inoculum.
• Use sap inoculum immediately after grinding.
• Gently rub the leaves to prevent damage.
• Spray the inoculated plants with distilled water within a few minutes after inoculation.

### Problem 7

Inability to detect somatic gene editing (Steps 34 and 35).

### Potential solution

• Select leaves exhibiting severe symptoms 4–5 days post-infection for editing assays.
• Test multiple target sequences to evaluate editing efficiency.
• Verify the integrity of the CRISPR/Cas inserts in progeny virus genomes by RT-PCR.
• Confirm the expression of Cas and GFP proteins by protein gel blotting.
• Amplify and sequence the targeted gene locus to exclude DNA polymorphism.

### Problem 8

Inability to regenerate plants from virus-infected tissues (Steps 44).

### Potential solution

• Use leaf tissues at early stages of disease onset (e.g., 1 or 2 days after viral infection) if the explants necrotize during tissue culture.
• Reduce the concentrations of ribavirin or omit it altogether in the MS induction medium.
• Include a mock-infected explant control to ensure that the regeneration protocol works.
• Test other regeneration protocols with different media recipes and hormone concentrations.

### Problem 9

Regenerated plants do not contain targeted mutations (Steps 47).

### Potential solution

• Select leaf tissues with conspicuous viral symptoms as explants, and ensure that substantial levels of somatic editing are detected in virus-infected tissues (explants).
• Test several target sites for a given gene and select the most effective ones.
• Analyze later batches of regenerants (e.g., after 3 months), as shoots may regenerate earlier from uninfected cells than from virus-infected ones. Also, knocking-out certain morphogenetic genes may delay *in vitro* differentiation and regeneration.
• Review the literature to ascertain if the knockout mutant of your target gene is viable.

## Resource availability

### Lead contact

Further information and requests for resources and reagents should be directed to and will be fulfilled by the lead contact, Zhenghe Li (lizh@zju.edu.cn).

### Technical contact

Technical questions on executing this protocol should be directed to and will be answered by the technical contact, Huanhuan Lou (892958808@qq.com).

### Materials availability

Plasmids generated in this study have been deposited to Addgene at https://www.addgene.org/Zhenghe_Li/ (#196286, 196291, 196292, 196293, 196295, 196296, and 196325–196329).

### Data and code availability

This study did not generate code or analyze datasets. There are no restrictions on data availability.

## Acknowledgments

This work was supported by National Key R&D Program of China grant no. 2022YFC2601000, FCTC grant no. 110202101034 (JY-11), and FYCTIC grant no. 2022JY03. C.Z. is supported by the Postdoctoral Fellowship Program of CPSF under grant number GZC20241527.

## Author contributions

Conceptualization, Z.L., Q.G., C.Z., and H.L.; experiment, H.L., C.Z., and H.X.; methodology, W.Z., J.J., J.Z., and L.X.; writing – original draft, H.L. and H.X.; writing – review and editing, H.L., H.X., C.Z., Q.G., Z.L., and the rest of the authors.

## Declaration of interests

The authors declare no competing interests.
